# Life Cycle Assessment on Wave and Tidal Energy Systems: A Review of Current Methodological Practice

**DOI:** 10.3390/ijerph17051604

**Published:** 2020-03-02

**Authors:** Xizhuo Zhang, Longfei Zhang, Yujun Yuan, Qiang Zhai

**Affiliations:** Department of Mechanical Engineering, School of Mechanical, Electrical & Information Engineering, Shandong University, Weihai 264209, China

**Keywords:** wave energy, tidal current energy, life cycle assessment, ISO

## Abstract

Recent decades have witnessed wave and tidal energy technology receiving considerable attention because of their low carbon emissions during electricity production. However, indirect emissions from their entire life cycle should not be ignored. Therefore, life cycle assessment (LCA) has been widely applied as a useful approach to systematically evaluate the environmental performance of wave and tidal energy technologies. This study reviews recent LCA studies on wave and tidal energy systems for stakeholders to understand current status of methodological practice and associated inherent limitations and reveal future research needs for application of LCA on wave and tidal technologies. The conformance of the selected LCAs to ISO 14040 (2006) and 14044 (2006) are critically analyzed in strict accordance with the ISO stepwise methodologies, namely, goal and scope definition, life cycle inventory (LCI) analysis, as well as life cycle impact assessment (LCIA). Our systematically screening of these studies indicates that few of the selected studies are of strict conformance with ISO 14040 and 14044 standards, which makes the results unreliable and thus further reduces the confidence of interested stakeholders. Further, our review indicates that current LCA practice on wave and tidal energies is lacking consideration of temporal variations, which should be addressed in future research, as it causes inaccuracy and uncertainties.

## 1. Introduction

Life cycle assessment (LCA) has been widely recognized as an efficient approach to evaluate the life cycle environmental impacts of a product or service by comprehensively encompassing all processes and environmental releases for specific environmental impact categories. As the most significant contributor to climate change, life cycle greenhouse gas (GHG) emission has been adopted by laws and regulations as an indicator to evaluate the environmental performance of clean energies. For instance, U.S. Energy Independence and Security Act of 2007, Section 526 [1] requires that life cycle GHGs for nonconventional petroleum sources must be less than or equal to such emissions from the equivalent conventional fuel produced from fossil sources. The early incorporation of environmental issues has been requested by the EU Strategic Environmental Assessment procedure (Directive 2001/42/EC) [2]. Declaration of life cycle GHG emissions has been required by current and future environmental regulations [3,4]. Among viable techniques for environmental assessment, LCA is a comprehensive stepwise method, including goal and scope definition, life cycle inventory (LCI) analysis, life cycle impact assessment (LCIA) and interpretation. As a technique of environmental management, the principles, framework, requirements and guidelines are suggested in ISO 14040 (2006) [5] and ISO 14044 [6]. As for wave and tidal energy, although recent decades have witnessed the emergence of new technologies, only a few wave and tidal systems are studied by LCA methods, because the technologies are still at such an early stage that limited funding is available for supporting research beyond technology development [7]. 

In recent decades, a few researchers have overviewed the LCAs on ocean energy systems. Banerjee et al. discuss the emission characteristics and energy accounting of wave and tidal energy systems for LCAs [8]. A comprehensive review of the current state of the art of research in the field of ocean energy systems, with an emphasis on research beyond technology or technological improvements is presented in [7]. Paredes et al. systematically evaluated the LCA studies of ocean energy technologies and presented a summary of the LCA results [9]. To our knowledge, there is no review work has been reported on a comprehensive and in-depth analysis of the methods adopted by the published LCAs on wave and tidal energy systems. In this study, we conduct an extensive review of recent LCAs on wave and tidal energy systems, with the following purposes: (a) summarizing the current status of the methodological practice; (b) identifying the limitations of methods of LCAs on wave and tidal energies; (c) revealing future research needs for wave and tidal LCAs from the methodological perspective.

## 2. Methodology

### 2.1. Literature Search Strategy

The selected international databases included: Web of Science Core Collection, BIOSIS Previews, Chinese Science Citation Database, Inspec, KCI-Korean Journal Database, MEDLINE, Russian Science Citation Index and SciELO Citation Index. Search keywords for topics and titles included: “ocean energy”, or “marine energy”, or “marine current energy”, or “ocean thermal energy”, or “salinity gradient energy”, or “wave energy”, or “wave power”, or “tidal energy”, or “tidal power”, or “tidal current”, or “tidal stream”, or “sea turbine”, or “wave energy conversion”, or “wave energy converter”, or “WEC”, or “tidal stream/barrage device”, or “tidal current turbine”; and “life cycle assessment”, or “LCA”, or “life cycle analysis”, or “environmental assessment”, or “environmental impact”, or “global warming”, or “greenhouse gas”, or “GHG”, or “carbon footprint”, or “carbon dioxide”, or “CO_2_”, or “embodied carbon”, or “carbon intensity”, or “CO_2_ intensity”, or “carbon audit”, or “carbon emission”, or “energy audit”, or “energy accounting”, or “energy intensity”, or “embodied energy”.

### 2.2. Case Studies Refining

Through the application of the above-mentioned keywords in Section 2.1, the search returned 1214 references. The found research literature was then further screened by applying such criteria as:

a) the studies are put together in English;
b) the studies are peer-reviewed journal articles, full conference papers excluding abstracts and posters, theses or dissertations, or official governmental reports;
c) the wave or tidal technologies are designed for production of electricity.

This finally left 18 studies, amongst which 4 cases were about tidal current energies [9,10,11,12,13], 12 cases were about wave energies [3,4,14,15,16,17,18,19,20,21,22,23] and 2 cases were about both tidal and wave energies [24,25]. 

#### 2.2.1. Spatial Distribution of Studied Wave and Tidal Energy Systems 

Geographical distribution of the selected LCA studies, as shown in Table 1, indicates that 11 systems are installed in European seas [3,9,11,13,14,15,16,17,18,19,22,23], 1 in New Zealand [12], 1 in China [20] and 1 in multicontinental locations [22]; three are located at hypothetical offshore locations [4,21,24].

**Figure 1:**
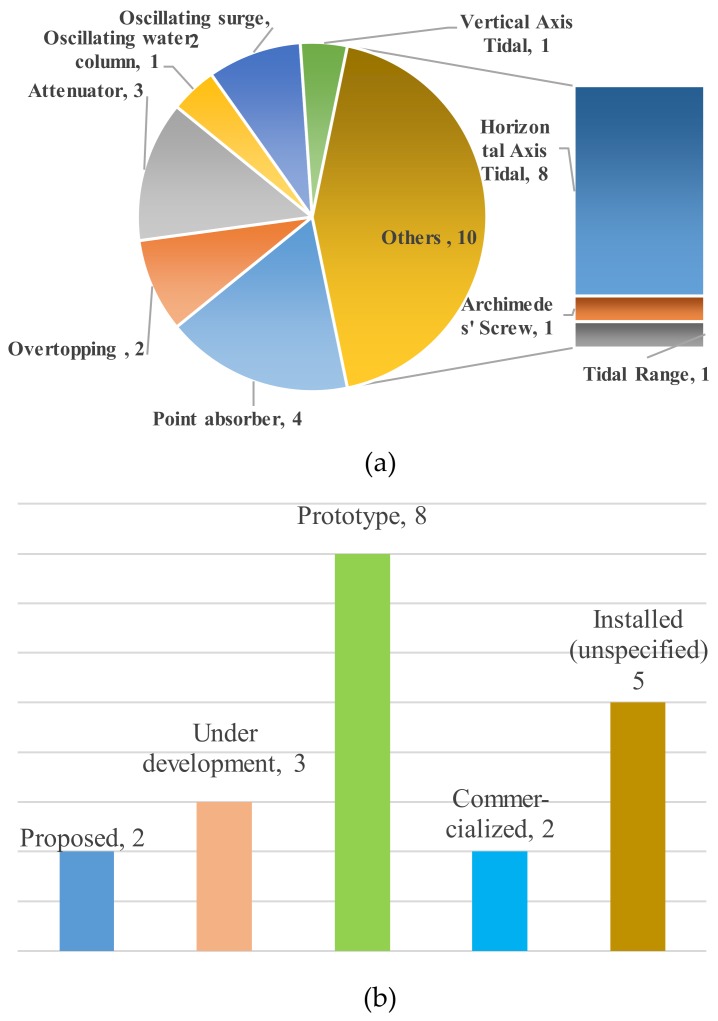
Technological coverage and development status of studied wave and tidal power systems. (a) Technological coverage; (b) Development status. Note: the number following each item in (a) indicates the number of the systems by specific type of technology; the number following each item in (b) indicates the number of the systems under the specific development status.

As shown in Figure 1, the selected literature studied two oscillating surge WECs [22,25], one oscillating water column [21], three attenuators [14,15,16,17], two overtopping [3,23], four point absorbers [4,19,20,22], one vertical axis tidal [9], eight horizontal axis tidal [11,12,13,25], one Archimedes [13] and one tidal range [25].

#### 2.2.2. Installed Capacities and Technological Development Status

Installed capacities of the studied systems range from 3 kW to 265.5 MW, as shown in Table 1. Only five studies take the capacity factors of the power generation systems for consideration [11,12,17,20,24]. As Figure 1b demonstrates, two systems are under proposal [12,24]; three are under development [4,21,22]; eight have prototypes installed and tested [3,13,14,19,20,23]; two are commercialized [15,17]; five are claimed being installed as of writing of the papers or reports, however with no further details provided regarding the installation, e.g., it is unknown whether they are pioneer plants or full-scale commercialized systems [9,11,13,18,25].

#### 2.2.3. ISO 14040 and 14044 Conformance Declarations

Currently, ideal practice of LCA is to follow the principles, framework, requirements and guidelines by international standards ISO 14040 (2006a) and 14044 (2006). Our review of the selected literature shows that only five studies claim that they followed ISO 14040 [11,14,15,20,21], two studies claimed that they followed ISO 14044 [12,17], and two claimed that they followed both ISO 14040 and 14044 [4,13].

## 3. Results

### 3.1. Goal and Scope

Definition of goal and scope is the first step of an LCA study, as it defines the purpose or application of the study, as well as the scope of assessment to be conducted. As per our analysis of the selected studies, energy and carbon are the most considered environmental indicators, so some LCA studies merely investigate the life cycle primary energy and carbon of the wave and tidal energy systems [11,12,13,14,18,19,23]. In this case, these studies are merely LCI studies, as shown in Table 1, since no life cycle impacts are assessed and discussed.

LCI results are normally calculated and interpreted through such indicators as energy intensity and carbon intensity, as well as energy and carbon payback. Inclusion of LCIA in the LCA studies can comprehensively illustrate the life cycle environmental performance of the studied systems, thus various levels of LCIA were conducted to further reveal the environmental impacts of the wave and tidal systems beyond energy consumption and carbon emission [3,4,9,15,16,17,20,21,22,24,25]. 

#### 3.1.1. Internal and External Application

Internal application of LCA is to identify the most significant contributors such as materials, processes and life cycle stages of ocean energy devices in terms of environmental impacts [11,24] by investigating the associated with life cycle stages [9]. The results can be a reference in identifying system improvement potentials [4,15,20] through choice of substitute materials and processes [4]. LCA results are widely applied for comparison between different wave and tidal energy systems as well as other renewable energy technologies [12,19,20,24]. 

#### 3.1.2. System Boundary

System boundary determines physical inclusion of materials and processes, temporal inclusion of long- and short-term releasements, as well as geographical factors into the product system for the LCA study. Wave and tidal energy systems are usually divided into such stages as material extraction, manufacturing, installation, operation and maintenance, decommission and disposal. As shown in Table 2, most of the analyzed studies adopt cradle-to-grave boundaries, i.e., include all the above-mentioned life cycle stages in the power system for LCA studies [3,4,9,10,11,12,13,14,15,16,17,18,19,20,21,24,25]. Only two studies apply cradle-to-gate system boundaries and exclude the stages of installation, operation and maintenance, decommission and disposal [22,23].

#### 3.1.3. Functional Unit

Functional unit enables that results from different LCA studies can be compared for product systems with similar functions [5]. As illustrated in Table 2, for wave and tidal energy systems, the main function is electricity production; therefore, the majority of the selected LCA studies define the functional unit as 1 kWh electricity generated [3,4,12,14,15,16,17,21,24,25]. Some studies further specify that the 1 kWh electricity is generated and fed to the national grid [4,24,25]. Few studies [11,18,19] do not claim functional unit definitions, however, they use per-kWh electricity for calculation of energy and carbon intensities. On this point then, 1 kWh electricity is the virtual functional unit of these studies. Other studies define the functional units as the entire power systems [9,13,20,23], as they intend to conduct the LCAs for merely internal purpose. 

#### 3.1.4. Cut-off Criteria

Exhaustive inclusion of inputs and outputs of the system is neither possible, since a product life cycle contains too many materials and too much energy consumption associated with unit processes and emissions, nor necessary, as the goal of an LCA is normally defined to identify the most significant contributors to the environmental impacts. Definition of appropriate cut-off criteria is therefore necessary to exclude less important inputs and outputs, with setting up percentages of mass, energy or environmental significance [6].

For wave and tidal systems, Table 2 shows that for inputs, energy flow and environmental significance are the most commonly used cut-off criteria [11,12,14,15,16,17], as embodied energy and carbon are the most relevant indicators. Only two of the selected LCA studies considered mass as the cut-off criteria [13,20]. Mass, energy and environmental significance are taken into consideration for cutting off by Dahlsten et al., 2009. As for outputs, two studies use mass and energy flow as the cut-off criteria [11,17], two use mass [13,20], and one uses mass, energy and environmental significance [4]. Among the selected LCA studies, only one of them defined environmental significance as the single cut-off criterion [23]. None of the above-mentioned criteria were described within eight of the selected studies [3,9,18,19,21,22,24,25].

#### 3.1.5. Allocation

Often different systems share inputs and outputs, thus, dividing and assigning them in between these systems is critical to ensure the accuracy of LCA studies [5]. There are different approaches for allocation in LCA practice, such as partitioning approach and substitutional approach. Partitioning approach is also called allocation in the sense of the word, which is based on the physical characteristics such as mass, volume and energy content. Through the application of substitutional approach, the burden of some byproducts of the product system is included into the system boundary, which means the burden of these byproducts is avoided when they enter the boundaries of other systems. Partitioning approach is adopted by most of the reviewed articles. As shown in Table 2, open-loop method is applied by some wave and tidal LCA studies, which defines recycling rate for materials (e.g., metallic materials) for foreground and background data [4,13,14,15,16,17]. Other studies described various recycling and reuse rates of materials, which are defined as closed-loop procedure [9,10,11,12,18,19,20,21,24,25]. In fact, mass and energy flow is so complicated that single open- or closed-loop does not always sufficiently describe the actual product system. Within this context, a combined or hybrid open and closed loop is more appropriate for the allocation modeling [13]. Two of the selected LCA studies did not describe their allocation procedure for their system modeling [22,23].

#### 3.1.6. Impact Categories Definition 

The definition of impact categories depends on the goal and scope definition and provides the range of interested environmental issues either from midpoint or endpoint perspective. The selected studies containing LCIAs all discuss the selection of impact categories in the scope definition [3,4,9,15,16,17,20,21,22].

#### 3.1.7. Critical Review

A critical review by experts certifies the validation of the LCA method, data collection and calculation and rationality of the interpretation [6]. None of the selected studies provides information regarding expert reviews [3,4,9,10,11,12,13,14,15,16,17,18,19,20,21,22,23,24,25].

### 3.2. Life Cycle Inventory Analysis

#### 3.2.1. Data Collection and Data Quality

As shown in Table 3, as per analyzed results of the selected LCA studies, the data for LCI are divided into three groups: primary data, which concern the foreground system; secondary data, which concern the background system; and unavailable data. For the reviewed studies, primary data are mainly collected from designer, developer and manufacturer [3,9,10,11,12,13,15,17,18,19,20,21,24,25]. The Inventory of Carbon and Energy (ICE), a database developed by the University of Bath is adopted as an important primary data source by some researchers [11,14,19]. It is also the case that primary data are based on calculation by the researchers [4,23]. 

Databases are widely used as a main source for secondary data collection. Commonly used databases include Ecoinvent [4,14,15,17,20,21,23,25], Gabi [24], EDIP [3], ETH-ESU 1996, ETH 1996, IDEMAT 2001 and BUWAL 1996 [9]. Literature is another important source for secondary data collection, which includes journals, conference papers, theses and previous LCA studies [3,11,12,13,14,18,21,25]. However, two of the studies do not describe the source of secondary data [19,22]. For unavailable data gaps existing for almost all wave and tidal LCA studies, reasonable assumptions are usually made for inputs and outputs of LCI [3,4,9,10,11,12,13,14,15,16,17,18,19,20,21,22,23,24,25].

#### 3.2.2. Data Calculation and Energy Flows

Our analysis shows that sources of fuels and electricity are considered by some studies [3,4,11,12,13,14,15,16,17,20,23,24,25]. A few studies and do not claim the sources of fuels and electricity [9,18,19,21,22]. None of the selected LCA studies discuss efficiency of conversion and distribution of energy flow. Among the studies considering the different fuels and electricity sources, most of them also describe the inputs and outputs associated with generation and use of that energy flow, except for three cases [11,12,17].

#### 3.2.3. Validation of Data

**Figure 2:**
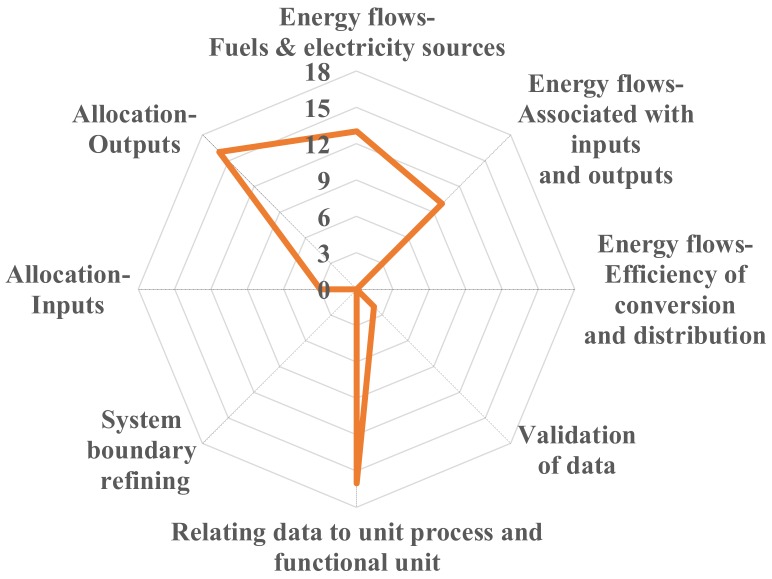
Life cycle inventory analysis of studied wave and tidal power systems. Note: the value of each item indicates the number of LCA studies that applied the specific method required in ISOs.

Validation of data can be performed by establishing balances of mass and energy or by analyzing release factors. Figure 2 indicates that only two of the selected LCA studies [19,21] describe the conservation of mass flow for the collected data. However, no further details about the data validation process were provided. Other studies do not provide information about the data validation procedure via either mass or energy conservation. 

#### 3.2.4. Relating of Data to Unit Process and Reference Flow of the Functional Unit

The relating of data to unit process and reference flow of the functional unit is an optional step of LCI. As shown in Figure 2, most of the selected LCA studies performed the relating of data to unit process and functional unit [3,4,11,12,13,14,15,16,17,18,19,20,21,22,23,24,25]. Two studies did not provide such information [3,9]. None of the selected LCA studies performed or provided information for the system refining based on the data processing. 

#### 3.2.5. Allocation of Inputs and Outputs

An allocation procedure is suggested by ISO since most industrial processes yield multiple, rather than single, outputs and are based on complicated material and energy inputs. As shown in Figure 2, the allocation procedures for reuse and recycling are described by the selected LCA studies except for two [22,23]. Allocation procedures for inputs were considered by few studies [12,17,25].

### 3.3. Life Cycle Impact Assessment

LCIA was conducted on the basis of inventory analysis results by means that the LCI results are assigned, characterized, normalized and weighted with application of given impact categories. Midpoint and endpoint methods look at different stages of the environmental impacts. LCI results are identified and assigned into appropriate impact categories per their environmental relevance and then characterized with specific category indicators so that the specific impact categories can be quantitatively interpreted. A midpoint impact category refers to an impact that contributes to specific aspects of human heath, natural environment or resources. [5]. It is mostly the case that the intended audience decide whether midpoint or endpoint level of environmental impacts should be assessed. As shown in Table 4, midpoint method is commonly adopted for current LCA studies on wave and tidal energy systems [3,4,9,15,16,17,20,21,22,23,24,25] except for one study [9].

Selected LCAs on wave and tidal energy systems conducted the classification and characterization requirements by ISO 14040 and 14044 via different methods such as Eco-indicator 99 [9], EDIP1997 [3], EDIP 2003 [15,16], ReCiPe [17,20,25], CED [17], PCR [4], CML [21] and ILCD [24]. Normalization is applied to illustrate environmental impact scores by comparison with reference scenarios, such as Europe scenario [9] and Atlantic base scenario [21]. As shown in Table 5, the most relevant impact category is climate change impact, followed by ecotoxicity, resource depletion, human toxicity, eutrophication, ozone layer and acidification. Other impacts such as radiation, particular matter formation, photochemical oxidant formation, bulk waste, land use, slags/ashes and hazardous waste are studied by few case studies. The most significant contributor to each investigated impact category is listed in Table 5.

## 4. Critical Discussion

Our analyses show that most of current LCA practices on wave and tidal systems are not commendably following the framework, guidelines and requirements established by ISO 14040 and 14044, although ISO standards make the results more convincing to the intended audience. Also, LCA results can be used for formal or official legal disclaimers only if ISO standards are well followed, as regulators and governments commonly count on them for safety insurance in most cases. Finally, results of different LCA studies can only be effectively compared if conducted with same or similar methodological standards.

It is noticed that conventional ISO LCA does not take into account the temporal variability of the inventory data, which is one of the recognized limitations [26,27,28,29,30,31,32,33]. Current LCA practice treats energy, materials, resources and emissions by means of linear summation [33,34,35,36]. Thus, various emissions of a material generated at different time periods are treated as a single aggregated emission generated at one time during the life cycle [37]. Apparently, this result is not accurate, as there never exists aggregated emission amount in real world cases [37]. Within this context, recent years have seen development of dynamic life cycle assessment (DLCA) by considering temporal dimension and applying different mathematical models [38]. As pointed out by Müller et al., DLCA shows different results of environmental impacts compared with conventional LCA, especially in climate change and toxicity [39]. 

Due to absence of commonly recognized mathematical method, the development of DLCA is still at its early stage [40]. Current DLCA application focuses on buildings [41,42,43,44,45,46] (Batouli and Mostafavi, 2017; Negishi et al., 2018; Hu, 2018; Su et al., 2019; Keiron et al., 2018; Bixler et al., 2019; Cardellini et al., 2018), transportation systems [47,48] and energy systems [49,50,51,52] because of their longevities. 

Thus, for wave and tidal energy systems, as systems with long lifespans (normally ≥20 years), application of DLCA will help reduce the inaccuracy and uncertainties of environmental impact results. Development of appropriate mathematical methods is encouraged for the conduction of DLCA.

## 5. Conclusions

The scope of this review includes a stepwise check of the selected LCAs on wave and tidal energy systems, from the perspective of their conformance with the ISO 14040 and 14044. The results show that the reviewed studies are carried out in accordance with the ISO standards at different levels. Non-strict conformance with the ISO standards weakens the reliability of the assessment results, whether they are purposed for internal or external applications. This further decreases the comparability between different wave and tidal energy systems, as well as with other energy technologies. Finally, the performed review illustrates that ignorance of temporal variation caused inaccuracy and uncertainty which should be addressed in future research.

## Figures and Tables

**Table 1:** General info of the studied wave and tidal systems studied by life cycle assessments (LCAs).

Reference	Device	Region	Installed capacity	Capacity factor	ISO Smartcards	
					14040	14044
Cavallaro et al. (2007)	Kobold	Italy	160 kW	N.A.	-	-
Douglas et al. (2008)	Seagen	UK	1.2 MW	48%	√	-
Rule et al. (2009)	Kaipara Harbor	New Zealand	200 MW	37%	-	√
Howell et al. (2013)	DeepGen; OpenHydro; ScotRenewables SR2000; Flumill	UK	1 MW; 2 MW; 2 MW; 2 MW	N.A.	√	√
Hans et al. (2007)	Wave Dragon	Denmark	7 MW	N.A.	-	-
Parker et al. (2007)	Pelamis	UK	750 kW	N.A.	√	-
Thomson et al. (2011a)	Pelamis	UK	750 kW	N.A.	√	-
Thomson et al. (2011b)	Pelamis	UK	750 kW	N.A.	√	-
Thomson et al. (2019)	Pelamis	UK	750 kW	45%	-	√
Dahlsten et al. (2009)	Seabased	Hypothetical	20 MW	N.A.	√	√
Walker et al. (2011)	Oyster	UK	315 kW	N.A.	-	-
Ombach et al. (2014)	Wave Star	Denmark	1000 kW	N.A.	14000	-
Zhai et al. (2018)	BRD	China	10 kW	50%	√	-
Elginoz et al. (2017)	MUP farm	Hypothetical	265.5 MW	Various	√	-
Curto et al. (2018)	DEIM	Italy	30 kW	N.A.	√	√
Patrizi et al. (2019)	OBREC	Italy	3 kW	N.A.	-	-
Uihlein et al. (2016)	Various	Unspecified	500kW-1 MW	34%; 0%	-	-
Douziech et al. (2016)	Annapolis; SeaGen; HS1000; HydraTidal; Oyster800	Canada; Ireland; UK; Norway; UK	20 MW; 1200 kW; 1000 kW; 1500 kW; 800 kW	N.A.	-	-

**Table 2:** Goal and scope definition of studied LCAs.

Reference	Study Type			Goal		Scope											
	LCA	LCI	CS	Int.	Ext.	System Boundary	Functional Unit	Cut-off Criteria						Allocation		ICD	CRD
								Inputs			Outputs			Open Loop	Closed Loop		
								M	E	ES	M	E	ES				
**Cavallaro et al. (2007)**	**√**	**-**	**-**	**√**	**-**	**X-grave**	**System**	**-**	**-**	**-**	-	-	-	-	√	√	-
Douglas et al. (2008)	-	√	√	√	-	X-grave	Undefined^1^	-	√	√	-	√	√	-	√	N.A.	-
Rule et al. (2009)	-	√	√	-	√	X-grave	1 kWh	-	√	√	-	-	-	-	√^2^	N.A.	-
Howell et al. (2013)		√	√	√		X-grave	System	√	-	-	√	-	-	√^3^		N.A.	-
Hans et al. (2007)	√		√	-	√	X-grave	1 kWh	-	-	-	-	-	-	√^4^	√^4^	√	-
Parker et al. (2007)		√	√	√		X-grave	1 kWh	-	√	√	-	-	-	√^3^	-	N.A.	-
Thomson et al. (2011a)	√	-	√	√	√	X-grave	1 kWh	-	√	√	-	-	-	√^5^	-	√	-
Thomson et al. (2011b)	√	-	-	√	√	X-grave	1 kWh	-	√	√	-	-	-	√^5^	-	√	-
Thomson et al. (2019)	√	-	√	√	-	X-grave	1 kWh	-	√	√	-	√	√	√^5^	-	√	-
Dahlsten et al. (2009)	√	-	-	√	-	X-grave	1 kWh	√	√	√	√	√	√	√^6^	-	√	-
Walker et al. (2011)	-	√	-	√	-	X-grave	Undefined	-	-	-	-	-	-	-	√^7^	N.A.	-
Ombach et al. (2014)	-	√	√	√	-	X-grave	Undefined	-	-	-	-	-	-	-	√^8^	N.A.	-
Zhai et al. (2018)	√	-	√	√	-	X-grave	System	√	-	-	√	-	-	-	√	√	-
Elginoz et al. (2017)	√	-	√	√	√	X-grave	1 kWh	-	-	-	-	-	-	-	√^9^	√	-
Curto et al. (2018)	√	-	√	√	-	X-gate	System	-	-	-	-	-	-	-	-	√	-
Patrizi et al. (2019)	-	√	-	√	-	X-gate	System	-	-	√	-	-	√	-	-	√	-
Uihlein et al. (2016)	√	-	√	√	-	X-grave	1 kWh	-	-	-	-	-	-	-	√^10^	√	-
Douziech et al. (2016)	√	-	√	-	√	X-grave	1 kWh	-	-	-	-	-	-	-	√^11^	√	-

Abbreviations: CS, comparative study; Int., internal; Ext., external; X, cradle; ICD, impact category definition; CRD, critical review definition; M, materials; E, Energy; ES, environmental significance; √, yes; -, not reported; N.A., not applicable. Notes: ^1^/kWh used for energy and carbon intensities; ^2^No recycling for disposal of field equip., reuse of half of turbines. ^3^Recycling only for steel. ^4^Steel, copper, aluminum, bronze, plastics used for other processes; concrete reused for road construction. ^5^Recycled content approach for both foreground and background processes. ^6^Polluter pays (EPD); no allocation for foreground data. ^7^Replacing primary material with recycled material in future. ^8^Recycling rate for metals is 90%, some materials not recycled, and instead incinerated or taken to land fill. ^9^Ninty percent recycling for metals, otherwise incineration or landfill. ^10^Various recycling rate for ferrous and nonferrous metals. ^11^ISO/TS 14067 closed-loop procedure.

**Table 3:** Data collection sources for studied LCAs.

Reference	Data Collection		
	Primary (Specific) Data	Secondary (Generic) Data	Unavailable Data
Cavallaro et al. (2007)	Designer	ETH-ESU 1996, IDEMAT 2001, BUWAL 1996, ETH-ESU 1996, IDEMAT 2001, and ETH 1996	Assumptions
Douglas et al. (2008)	MCT (designer/manufacturer); Inventory of Carbon and Energy (ICE, a database by the University of Bath);	Literature (existing LCAs, journals, and textbooks)	Assumptions
Rule et al. (2009)	Reports regarding the studied systems	Literature	Assumptions
Howell et al. (2013)	Manufacturers, brochures and presentations	Literature	Assumptions
Hans et al. (2007)	Designer and Manufacturer	EDIP database; literature (existing LCAs and reports)	Assumptions
Parker et al. (2007)	Inventory of Carbon and Energy (ICE)	Ecoinvent database; literature (journals, conference papers and previous LCA studies)	Assumptions
Thomson et al. (2011a)	Manufacturer	Ecoinvent database	Assumptions
Thomson et al. (2011b)	Manufacturer	Ecoinvent database	Assumptions
Thomson et al. (2019)	PWP’s own records by Parker et al.	Ecoinvent database	Assumptions
Dahlsten et al. (2009)	Calculation based on drawing, product sheets, product specific processes	Ecoinvent;	Assumptions
Walker et al. (2011)	Company website, device patent, installation contractor, and EMEC	Literature	Assumptions
Ombach et al. (2014)	Designer, ICE database, compiled by the University of Bath	Unspecified	Assumptions
Zhai et al. (2018)	Designer	Ecoinvent database	Assumptions
Elginoz et al. (2017)	Designer	Ecoinvent; literature (reports, thesis, scientific papers)	Assumptions
Curto et al. (2018)	Unspecified	Unspecified	Assumptions
Patrizi et al. (2019)	Metric computations	Ecoinvent database	Assumptions
Uihlein et al. (2016)	JRC ocean energy database	GaBi database	Assumptions
Douziech et al. (2016)	Plant developers	Ecoinvent; literature	Assumptions

**Table 4:** Life cycle impact assessment (LCIA) of studied LCAs.

Reference	Method, Midpoint/End-point	Classification brk (Assignment of LCI Results)	Characterization brk (Calculation of Indicator Results)	Normalization/Reference (Optional)	Weighting (Optional)
**Cavallaro et al. (2007)**	**Eco-indicator 99, midpoint and endpoint**	**√**	**Unspecified, no details presented**	**Yes/Europe**	-
Hans et al. (2007)	EDIP1997, midpoint	√	Unspecified, no details presented	Yes/Unspecified	-
Thomson et al. (2011a)	EDIP 2003, midpoint	√	√	-	-
Thomson et al. (2011b)	EDIP 2003, midpoint	√	√	-	-
Thomson et al. (2019)	ReCiPe and CED, midpoint	√	√	-	-
Dahlsten et al. (2009)	PCR, midpoint	√	√	-	-
Zhai et al. (2018)	ReCiPe, midpoint	√	√	-	-
Elginoz et al. (2017)	CML 2001, midpoint	√	√	Yes/Atlantic base scenario	-
Curto et al. (2018)	Unspecified, midpoint	√	√	-	-
Patrizi et al. (2019)	Unspecified, midpoint	√	√	-	-
Uihlein et al. (2016)	ILCD, midpoint	√	√	-	-
Douziech et al. (2016)	ReCiPe, midpoint	√	√	-	-

**Table 5:** Life cycle impact categories and association with most significant contributors.

Reference	Impact categories													
	CC	OD	EXT	ACD	EUT	HT	HW	RD	POF	SA	RD	LU	PMF	BW
[3]	√^1^	√^1^	√^1^	√^1^	√^1^	√^1^	√^1^	-	√^1^	√^1^	√^1^	-	-	√^1^
[4]	M	M	-	M	M	-	-	M	M	-	-	-	-	-
[10]	√^1^	√^1^	M	√^1^	√^1^	-	-	M	-	-	-	√^1^	√^1^	-
[15]	M	M	O&M	M	O&M	M	M	M	-	M	M	-	-	M
[16]	M	M	O&M	O&M	O&M	M	M	M	-	M	M	-	-	M
[17]	M	O&M	M	O&M	O&M	M	-	M	O&M	-	M	M	M	-
[20]	M	M	EOL	M	EOL	M	-	M	M	-	M	M	M	-
[21]	M	M	M	M	M	M	-	M	M	-	-	-	-	-
[22]	√^1^	-	√^1^	-	-	√^1^	-	√^1^	-	-	-	-	-	-
[23]	M	-	-	-	-	-	-	-	-	-	-	-	-	-
[24]	M	M	M	M	M	M	M	M	M	-	M	-	M	-
[25]	M	-	EOL	-	-	EOL	-	M	-	-	-	-	M	-

Notes and Abbreviations: √, investigated; ^1^ with no contributor specified; -, not investigated; M, manufacturing; O&M, operation and maintenance; EOL, end of life; CC, climate change; OD, ozone depletion; EXT, ecotoxicity; ACD, acidification; EUT, eutrophication; HT, human toxicity; HW, hazardous waste; RD, resource depletion; POF, photochemical oxidant formation; SA, slags/ashes; RD, radiation; LU, land use; PMF, particular matter formation; BW, bulk waste.

## References

[1] US Public Law 110–140, 110 Congress, Energy Independence and Security Act of 2007. https://www.congress.gov/110/plaws/publ140/PLAW-110publ140.pdf.

[2] Azzellino A., Lanfredi C., Contestabile P., Ferrante V., Vicinanza D. Strategic environmental assessment to evaluate WEC projects in the perspective of the environmental cost-benefit analysis. Proceedings of the Twenty-First International Offshore and Polar Engineering Conference.

[3] Hans C.S., Stefan N., Stefan A., Hauschild M.Z. (2007). Life Cycle Assessment of the Wave Energy Converter: Wave Dragon. https://backend.orbit.dtu.dk/ws/portalfiles/portal/3711218/WaveDragon.pdf.

[4] Dahlsten H. (2009). Life Cycle Assessment of Electricity from Wave Power. http://urn.kb.se/resolve?urn=urn:nbn:se:slu:epsilon-s-2115.

[5] ISO14040:2006 (2006). Environmental Management Life Cycle Assessment: Principle and Framework.

[6] ISO14044:2006 (2006). Environmental Management-Life Cycle Assessment: Requirements and Guidelines.

[7] Uihlein A., Magagna D. (2016). Wave and tidal current energy-A review of the current state of research beyond technology. Renew. Sustain. Energy Rev..

[8] Banerjee S., Duckers L., Blanchard R.E. (2013). An overview on green house gas emission characteristics and energy evaluation of ocean energy systems from life cycle assessment and energy accounting studies. J. Appl. Nat. Sci..

[9] Paredes M.G., Padilla-Rivera A., Güereca L.P. (2019). Life Cycle Assessment of Ocean Energy Technologies: A Systematic Review. J. Mar. Sci. Eng..

[10] Cavallaro F., Coiro D. Life Cycle Assessment (LCA) of a marine current turbine for cleaner energy production. Proceedings of the 3rd International Conference on Life Cycle Management.

[11] Douglas C.A., Harrison G.P., Chick J.P. (2008). Life cycle assessment of the Seagen marine current turbine. Proc. Inst. Mech. Eng. Part M J. Eng. Marit. Environ..

[12] Rule B.M., Worth Z.J., Boyle C. (2009). Comparison of Life Cycle Carbon Dioxide Emissions and Embodied Energy in Four Renewable Electricity Generation Technologies in New Zealand. Environ. Sci. Technol..

[13] Howell R.J., Walker S., Hodgson P., Griffin A. (2013). Tidal energy machines: A comparative life cycle assessment. Proc. Inst. Mech. Eng. Part M J. Eng. Marit. Environ..

[14] Parker R.P.M., Harrison G.P., Chick J.P. (2007). Energy and Carbon Audit of an Offshore Wave Energy Converter. Proc. Inst. Mech. Eng. Part A J. Power Energy.

[15] Thomson C., Harrison G., Chick J. Full Life Cycle Assessment of a Wave Energy Converter. Proceedings of the IET Renewable Power Generation Conference.

[16] Thomson R.C., Harrison G.P., Chick J. Life Cycle Assessment in the Marine Renewable Energy Sector. Proceedings of the LCA XI International Conference.

[17] Thomson R.C., Chick J., Harrison G. (2019). An LCA of the Pelamis wave energy converter. Int. J. Life Cycle Assess..

[18] Walker S., Howell R. (2011). Life cycle comparison of a wave and tidal energy device. Proc. Inst. Mech. Eng. Part M J. Eng. Marit. Environ..

[19] Ombach G. (2014). Design and safety considerations of interoperable wireless charging system for automotive. Int. Conf. Ecol. Veh. Renew. Energ..

[20] Zhai Q., Zhu L., Lu S. (2018). Life Cycle Assessment of a Buoy-Rope-Drum Wave Energy Converter. Energies.

[21] Elginoz N., Bas B. (2017). Life Cycle Assessment of a multi-use offshore platform: Combining wind and wave energy production. Ocean Eng..

[22] Curto D., Neugebauer S., Viola ATraverso M., Franzitta V., Trapanese M. First Life Cycle Impact Considerations of Two Wave Energy Converters. Proceedings of the Oceans conference.

[23] Patrizi N., Pulselli R.M., Neri E., Niccolucci V., Vicinanza D., Contestabile P., Bastianoni S. (2019). Lifecycle Environmental Impact Assessment of an Overtopping Wave Energy Converter Embedded in Breakwater Systems. Front. Energy Res..

[24] Uihlein A. (2016). Life cycle assessment of ocean energy technologies. Int. J. Life Cycle Assess..

[25] Douziech M., Hellweg S., Verones F. (2016). Are Wave and Tidal Energy Plants New Green Technologies?. Environ. Sci. Technol..

[26] Yuan C., Wang E., Zhai Q., Yang F. (2015). Temporal discounting in life cycle assessment: A critical review and theoretical framework. Environ. Impact Assess. Rev..

[27] Hellweg S., Frischknecht R. (2004). Evaluation of long-Term impacts in LCA. Int. J. Life Cycle Assess..

[28] Pinsonnault A., Lesage P., Levasseur A., Samson R. (2014). Temporal differentiation of background systems in LCA: Relevance of adding temporal information in LCI databases. Int. J. Life Cycle Assess..

[29] Potting J., Hauschild M. (2005). Background for Spatial Differentiation in Life Cycle Impact Assessment—The EDIP2003 Methodology. Environmental Project No. 996. http://www2.mst.dk/Udgiv/publications/2005/87–7614–581–6/html/indhold_eng.htm.

[30] Riva A., D′Angelosante S., Trebeschi C. (2006). Natural gas and the environmental results of life cycle assessment. Energy.

[31] Shah V.P., Ries R.J. (2009). A characterization model with spatial and temporal resolution for life cycle impact assessment of photochemical precursors in the United States. Int. J. Life Cycle Assess..

[32] Yuan C.Y., Simon R., Mady N., Dornfeld D. Embedded temporal difference in life cycle assessment: Case study on VW Golf A4 CAR. Proceedings of the IEEE International Symposium on Sustainable System & Technology.

[33] Zhai Q., Ciardo K., Yuan C.Y. Temporal discounting for life cycle assessment: Perspectives and mechanisms. Proceedings of the 17th CIRP International Conference on Life Cycle Engineering.

[34] Kendall A., Chang B., Sharpe B. (2009). Accounting for time-dependent effects in biofuel life cycle greenhouse gas emissions calculations. Environ. Sci. Technol..

[35] Reap J., Roman F., Duncan S., Bras B. (2008). A survey of unresolved problems in life cycle assessment. Part 1: Goal & scope and inventory analysis. Int. J. Life Cycle Assess..

[36] Huppes G. (2005). Methods for life cycle inventory of a product. J. Clean. Prod..

[37] Owens J.W. (1997). Life-Cycle assessment in relation to risk assessment: An evolving perspective. Risk Anal..

[38] Chen S., Sun Z., Li S., Liu Y., Shi X. (2018). Research and application status of dynamic life cycle assessment. China Environ. Sci..

[39] Müller A., Wörner P. (2019). Impact of dynamic CO2 emission factors for the public electricity supply on the life-Cycle assessment of energy efficient residential buildings. IOP Conf. Ser. Earth Environ. Sci..

[40] Su S., Li X., Zhu Y. (2019). Dynamic assessment elements and their prospective solutions in dynamic life cycle assessment of buildings. Build. Environ..

[41] Batouli M., Mostafavi A. (2017). Service and performance adjusted life cycle assessment: A methodology for dynamic assessment of environmental impacts in infrastructure systems. Sustain. Resilient Infrastruct..

[42] Negishi K., Tiruta-Barna L., Schiopu N., Lebert A., Chevalier J. (2018). An operational methodology for applying dynamic Life Cycle Assessment to buildings. Build. Environ..

[43] Hu M. (2018). Dynamic life cycle assessment integrating value choice and temporal factors-A case study of an elementary school. Energy Build..

[44] Keiron P., Roberts David A., Turner J., Anne M., Stringfellow Bello I., Powrie W., Watson G. (2018). SWIMS: A dynamic life cycle-Based optimisation and decision support tool for solid waste management. J. Clean. Prod..

[45] Bixler T.S., Houle J., Ballestero T.P., Mo W. (2019). A dynamic life cycle assessment of green infrastructures. Sci. Total Environ..

[46] Cardellini G., Mutel C.L., Vial E., Muys B. (2018). Temporalis, a generic method and tool for dynamic Life Cycle Assessment. Sci. Total Environ..

[47] Onat N.C., Kucukvar M., Tatari O. (2016). Uncertainty-Embedded dynamic life cycle sustainability assessment framework: An ex-Ante perspective on the impacts of alternative vehicle options. Energy.

[48] Onat N.C., Kucukvar M., Tatari O. (2016). Integration of system dynamics approach toward deepening and broadening the life cycle sustainability assessment framework: A case for electric vehicles. Int. J. Life Cycle Assess..

[49] Louis J., Pongracz E. (2017). Life cycle impact assessment of home energy management systems (HEMS) using dynamic emissions factors for electricity in Finland. Environ. Impact Assess. Rev..

[50] Beloin-Saint-Pierre D., Levasseur A., Margni M., Blanc I. (2016). Implementing a dynamic life cycle assessment methodology with a case study on domestic hot water production. J. Ind. Ecol..

[51] Zhang B., Chen B. (2016). Dynamic Hybrid Life Cycle Assessment of CO2 Emissions of a Typical Biogas Project. Energy Procedia.

[52] Pehnt M. (2006). Dynamic life cycle assessment (LCA) of renewable energy technologies. Renew. Energy.

